# Oral mouthwashes for asymptomatic to mildly symptomatic adults with COVID-19 and salivary viral load: a randomized, placebo-controlled, open-label clinical trial

**DOI:** 10.1186/s12903-024-04246-1

**Published:** 2024-04-25

**Authors:** Daisuke Onozuka, Satoko Takatera, Hiroo Matsuo, Hisao Yoshida, Shigeto Hamaguchi, Shungo Yamamoto, Ryuichi Minoda Sada, Koichiro Suzuki, Keiji Konishi, Satoshi Kutsuna

**Affiliations:** 1 Department of Oral Microbe Control, Graduate School of Medicine, Osaka University, 2-2 Yamadaoka, Osaka, 565-0871 Osaka Japan; 2 Department of Infection Control and Prevention, Graduate School of Medicine, Osaka University, 2-2 Yamadaoka, Osaka, 565-0871 Osaka Japan; 3 Department of Transformative Analysis for Human Specimen, Graduate School of Medicine, Osaka University, Osaka, Japan; 4 Division of Fostering Required Medical Human Resources, Center for Infectious Disease Education and Research (CiDER), Osaka University, Osaka, Japan; 5 Department of Transformative Protection to Infectious Disease, Graduate School of Medicine, Osaka University, Osaka, Japan; 6 The Research Foundation for Microbial Diseases of Osaka University (BIKEN), Osaka, Japan

**Keywords:** Cetylpyridinium chloride, COVID-19, Mouthwash, On-demand aqueous chlorine dioxide solution, Randomized clinical trial

## Abstract

**Background:**

Recent randomized clinical trials suggest that the effect of using cetylpyridinium chloride (CPC) mouthwashes on the severe acute respiratory syndrome coronavirus 2 (SARS-CoV-2) viral load in COVID-19 patients has been inconsistent. Additionally, no clinical study has investigated the effectiveness of on-demand aqueous chlorine dioxide mouthwash against COVID-19.

**Methods:**

We performed a randomized, placebo-controlled, open-label clinical trial to assess for any effects of using mouthwash on the salivary SARS-CoV-2 viral load among asymptomatic to mildly symptomatic adult COVID-19-positive patients. Patients were randomized to receive either 20 mL of 0.05% CPC, 10 mL of 0.01% on-demand aqueous chlorine dioxide, or 20 mL of placebo mouthwash (purified water) in a 1:1:1 ratio. The primary endpoint was the cycle threshold (Ct) values employed for SARS-CoV-2 salivary viral load estimation. We used linear mixed-effects models to assess for any effect of the mouthwashes on SARS-CoV-2 salivary viral load.

**Results:**

Of a total of 96 eligible participants enrolled from November 7, 2022, to January 19, 2023, 90 were accepted for the primary analysis. The use of 0.05% CPC mouthwash was not shown to be superior to placebo in change from baseline salivary Ct value at 30 min (difference vs. placebo, 0.640; 95% confidence interval [CI], -1.425 to 2.706; *P* = 0.543); 2 h (difference vs. placebo, 1.158; 95% CI, -0.797 to 3.112; *P* = 0.246); 4 h (difference vs. placebo, 1.283; 95% CI, -0.719 to 3.285; *P* = 0.209); 10 h (difference vs. placebo, 0.304; 95% CI, -1.777 to 2.385; *P* = 0.775); or 24 h (difference vs. placebo, 0.782; 95% CI, -1.195 to 2.759; *P* = 0.438). The use of 0.01% on-demand aqueous chlorine dioxide mouthwash was also not shown to be superior to placebo in change from baseline salivary Ct value at 30 min (difference vs. placebo, 0.905; 95% CI, -1.079 to 2.888; *P* = 0.371); 2 h (difference vs. placebo, 0.709; 95% CI, -1.275 to 2.693; *P* = 0.483); 4 h (difference vs. placebo, 0.220; 95% CI, -1.787 to 2.226; *P* = 0.830); 10 h (difference vs. placebo, 0.198; 95% CI, -1.901 to 2.296; *P* = 0.854); or 24 h (difference vs. placebo, 0.784; 95% CI, -1.236 to 2.804; *P* = 0.447).

**Conclusions:**

In asymptomatic to mildly symptomatic adults with COVID-19, compared to placebo, the use of 0.05% CPC and 0.01% on-demand aqueous chlorine dioxide mouthwash did not lead to a significant reduction in SARS-CoV-2 salivary viral load. Future studies of the efficacy of CPC and on-demand aqueous chlorine dioxide mouthwash on the viral viability of SARS-CoV-2 should be conducted using different specimen types and in multiple populations and settings.

**Supplementary Information:**

The online version contains supplementary material available at 10.1186/s12903-024-04246-1.

## Introduction

Since the sudden emergence of severe acute respiratory syndrome coronavirus 2 (SARS-CoV-2) in China, coronavirus disease 2019 (COVID-19) has had a global impact, with considerable morbidity, mortality, and economic burden [1]. As of 6 December 2023, the number of confirmed COVID-19 cases worldwide stood at 772,138,818, including 6,985,964 deaths [2].

The oral cavity is involved in COVID-19 infection. This implicates saliva and the salivary glands as potential sources of COVID-19 transmission [3–5]. Accordingly, mouthwashes containing substances with virucidal activity have been investigated to prevent COVID-19 infection and reduce viral spread [6]. Cetylpyridinium chloride (CPC) is commonly used as a bactericide in mouthwash, lozenges, and sprays [7]. A recent review of randomized controlled trials (RCTs) revealed that CPC mouthwash exerts definite virucidal activity against SARS-CoV-2 in saliva [8]. Other recent reviews have also shown a reduction in SARS-CoV-2 salivary viral load following the use of mouthwashes with CPC [9, 10] suggesting the potential for reducing the oropharyngeal load of SARS-CoV-2 [11]. Other RCTs suggested that CPC mouthwashes reduced viral infectivity [12, 13]. However, it has also been demonstrated that SARS-CoV-2 viral load showed no significant difference associated with CPC mouthwash use [14–16]. A recent meta-analysis has shown that CPC mouthwashes did not reduce the number of bacterial colony-forming units in dental aerosols [17, 18]. Although many of these studies demonstrated sufficient power, some studies were limited by decreased power due to small sample size [18, 19]. Additionally, mouthwash concentrations were heterogeneous between the studies. Although a previous experimental study suggested that lower concentrations of CPC such as 0.001–0.005% (10–50 µg/mL) may suppress infectivity [7], few clinical studies have evaluated the effects of 0.05% CPC mouthwash [16], and the clinical effectiveness of 0.05% CPC mouthwashes in reducing SARS-CoV-2 is still limited.

A mouthwash containing an on-demand aqueous chlorine dioxide solution (MA-T™; Japan MA-T Industrial Association, Tokyo, Japan) was recently developed to overcome the limitations of ordinary chlorine dioxide for human use [20, 21]. On-demand aqueous chlorine dioxide generates free radicals by catalytic action only when exposed to viruses or live bacteria in the respiratory system, and then exerts strong microbicidal activity [21, 22]. It has been suggested that on-demand aqueous chlorine dioxide may have potential as a disinfectant mouthwash [23]. However, no clinical study has investigated the effectiveness of on-demand aqueous chlorine dioxide mouthwash against COVID-19. Thus, further evaluation of this agent might lead to it being assigned a role in decreasing the salivary viral load of SARS-CoV-2.

Here, we conducted a clinical trial to investigate the associations of CPC and on-demand aqueous chlorine dioxide mouthwash use with SARS-CoV-2 salivary viral load for asymptomatic to mildly symptomatic adults with COVID-19 in Japan.

## Methods

### Overview of the trial

This open-label, randomized, placebo-controlled clinical trial was approved by the Ethical Committee of Osaka University (No. S22003) on 6/10/2022, and was registered with the Japan Registry of Clinical Trials (jRCT) (No. jRCTs051220107) on 18/10/2022. Patient registration was conducted from 7/11/2022 to 19/1/2023 in Osaka, Japan. We evaluated the viral load associated with the use of mouthwashes among asymptomatic to mildly symptomatic adult patients with COVID-19.

### Patients

Eligible patients were required to be ≥ 18 years old; have COVID-19 infection confirmed by nucleic-acid amplification tests (reverse transcription-polymerase chain reaction [RT-PCR], loop-mediated isothermal amplification [LAMP], or antigen tests) without or with mild symptoms of COVID-19 ≤ 7 days after onset; have been recuperating at a hotel in Osaka, Japan; have no clinical contraindication to mouthwash; and have access to smartphones with communication applications. Key exclusion criteria were a previously confirmed COVID-19 infection with or without hospitalization, an incipient need for hospitalization, current pregnancy/breastfeeding, and receipt since developing COVID-19 of antiviral or immunosuppressive medicines such as remdesivir, molnupiravir, nirmatrelvir/ritonavir, sotrovimab, casirivimab/imdevimab, anti-interleukin-6 receptor antibody, Janus kinase inhibitors, or corticosteroids. Collected data on COVID-19 patients included demographic data (sex and age); body mass index; smoking history; comorbidity; vaccination status; days from onset to diagnosis; and pulse oximetry (SpO_2_).

### Intervention

An interactive Web response system was employed to randomize eligible COVID-19 patients in a 1:1:1 ratio to receive and use a mouth rinse consisting of 20 mL of placebo (purified water) for 1 min, 20 mL of 0.05% CPC mouthwash for 30 s, or 10 mL of 0.01% on-demand aqueous chlorine dioxide mouthwash for 1 min. Purified water was used as placebo mouthwash, and was not similar in color or taste to any of the intervention agents. To minimize allocation bias, allocation concealment was performed with an interactive Web response system until randomization was finished on the system. Randomization was stratified according to symptoms presence or absence and to the number of days from onset (asymptomatic, within three days from onset, or four days or more from onset). The randomization list was masked to the investigators, study monitors, and laboratory personnel until the database was locked. CPC and on-demand aqueous chlorine dioxide mouthwashes were manufactured by Earth Corporation (Tokyo, Japan), and the placebo was obtained from Hikari Pharmaceutical Corporation (Tokyo, Japan). Because CPC mouthwash is colored and flavored, this trial was conducted under an open-label design.

### Outcomes

We used SARS-CoV-2 salivary viral load as measured by RT-PCR cycle threshold (Ct) values as the primary endpoint. Ct values reflect the number of RT-PCR cycles necessary to detect identifiable mRNA. The individuals produced unstimulated saliva samples after abstaining from eating, drinking, or performing oral hygiene activities for at least 30 min prior to collection. The baseline saliva sample was collected and immediately after this, patients rinsed their mouths with the assigned mouthwash/placebo. Six saliva samples were collected at baseline (immediately before breakfast [08:00]) and at 30 min (08:30), 2 h (10:00), 4 h (immediately before lunch [12:00]), 10 h (immediately before dinner [18:00]), and 24 h (immediately before breakfast in the next morning [08:00]) before the use of mouthwash/water. After taking saliva samples, all individuals were asked to newly rinse with the allocated mouthwash/water at baseline and at each of 4 h, 10 h, and 24 h. Saliva samples were collected in an individual sterile tube and stored at 4 °C until testing within 36 h of collection at the Research Foundation for Microbial Diseases of Osaka University (BIKEN).

### Sample collection and RT-PCR

Saliva samples were collected with an Aptima Multitest Swab Specimen Collection Kit (PRD-03546, Hologic, San Diego CA, USA) [24]. The swab was inserted into the mouth for 60 s, swept around the interior of the oral cavity, and then placed into a transport tube containing 2.9 mL of transport medium [25]. Specimens were transported on cool packs and stored at 4 °C for a maximum of 6 days. The results were detected by an RT-PCR technique with the primer and probe set of L452R (SARS-CoV-2) Ver.2 (RC346A, TAKARA Bio Inc. Shiga, Japan) [26]. Target-specific amplification methodology with fluorescent probes was used to identify mutation of the L452R of the SARS-CoV-2 spike (S) gene and the presence of an internal control-S (PRD-04332, Hologic, Bedford MA, USA) [27]. Following the manufacturer’s protocol, the transport tubes were loaded onto an analyzer (Panther Fusion System, Hologic) for nucleic acid extraction, and RT-PCR assays for the detection of SARS-CoV-2 RNA were performed [28, 29]. These laboratory-developed tests were based on real-time RT-PCR using a Panther Fusion System which was previously validated for use with saliva specimens [30, 31]. A Ct value of 45 (no identifiable mRNA after 45 RT-PCR cycles) was considered a negative real-time RT-PCR result.

### Sample size

A parallel, 3-group design (with one control group and 2 mouthwash groups) will be used to test whether the mean for each mouthwash group is different from the control group mean (H0: δ = 0 versus H1: δ ≠ 0, δ = µi - µc) [32–36]. The null hypothesis was that CPC/on-demand aqueous chlorine dioxide mouthwash would not reduce salivary Ct values compared with placebo. The hypotheses will be evaluated using 2 two-sided, two-sample, Bonferroni-adjusted, unequal-variance (Welch’s) t-tests, with an overall Type I error rate (α) of 0.05. The group standard deviations (beginning with the control group) are assumed to be 1, 1, and 1. The control group mean is assumed to be 0. Because a previous randomized controlled trial suggested that several types of mouthwash decreased SARS-CoV-2 salivary viral load by up to approximately 90% [37], the mean reduction in salivary viral load with the use of CPC/on-demand aqueous chlorine dioxide mouthwashes assumed to be the same. To detect the mouthwash means 0.9 and 0.9 with at least 80% power for each test, the (equal) group sample size needed for each of the 3 groups (control and mouthwashes) will be 25 (totaling 75 subjects). Anticipating a 20% dropout rate, group sizes of 32, 32, and 32 subjects should be enrolled to obtain final group sample sizes of 25, 25, and 25 subjects. Sample size calculations were performed with PASS 2023 (NCSS, LLC. Kaysville, Utah, USA) [38].

### Statistical analysis

The baseline characteristics of the subjects were described using number (%) and median [interquartile range (IQR)], as appropriate. Linear mixed-effects models were used to estimate the effects of CPC and on-demand aqueous chlorine dioxide mouthwash compared with placebo on the SARS-CoV-2 salivary viral load. We included the subject and time terms as random effects in the model. The estimated effects of CPC and on-demand aqueous chlorine dioxide mouthwash compared with placebo are presented as estimates with 95% confidence intervals (CIs).

To improve statistical efficiency and enhance the ability to correctly interpret the results, baseline characteristics in RCT design were adjusted as advised by the US Food and Drug Administration and the European Medicines Agency [39]. Thus, we conducted sensitivity analysis and adjusted for sex, age, body mass index, smoking history, comorbidity, COVID-19 vaccination, days from onset to diagnosis, and pulse oximetry (SpO_2_) in this model.

The statistical tests were two-tailed, and a value of *P* < 0.05 was considered to indicate statistical significance. All analyses were performed with Stata 18.0 (StataCorp LLC, College Station, TX, USA).

## Results

**Fig. 1:**
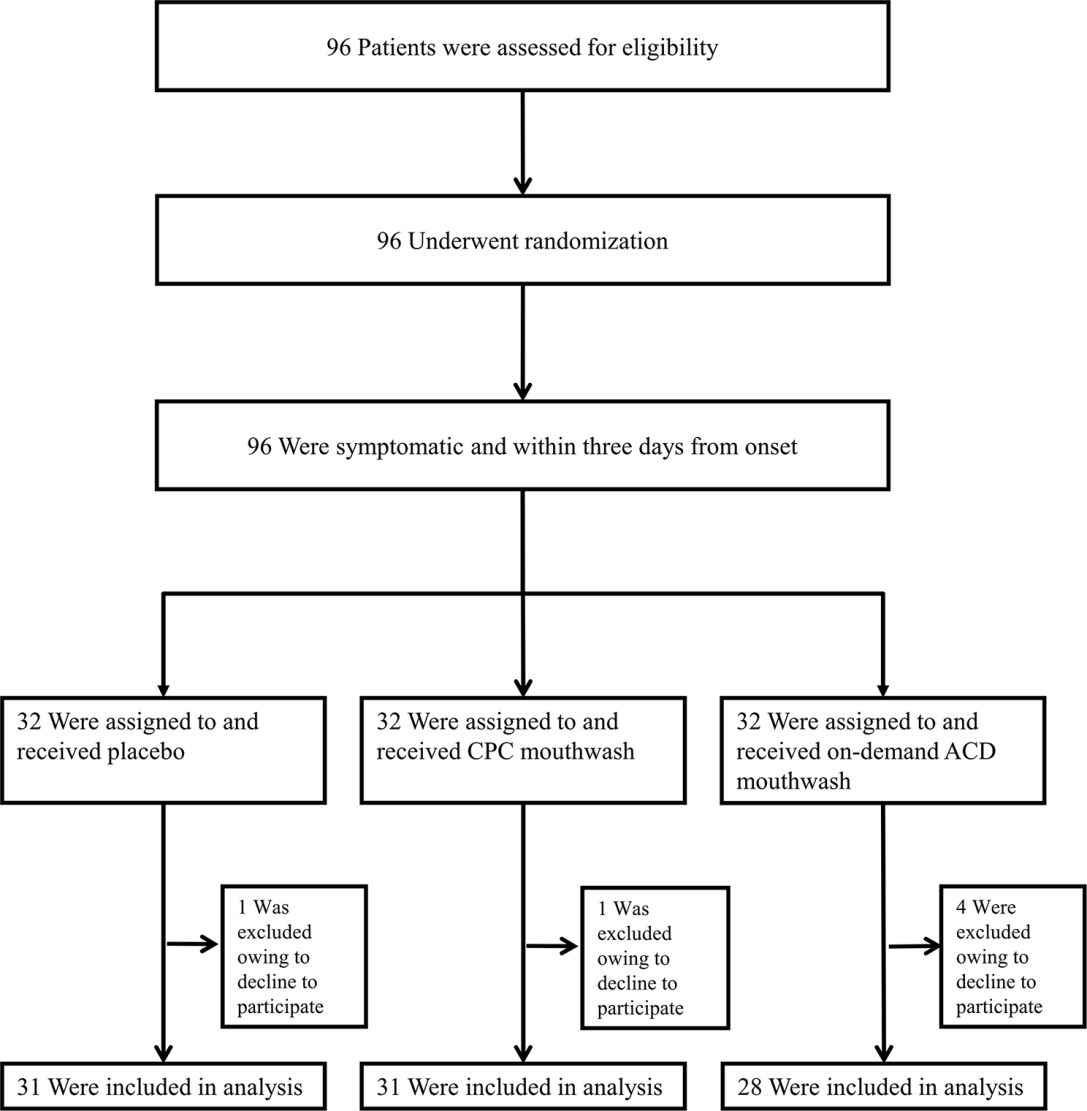
Participants’ data included in the study. ACD = aqueous chlorine dioxide; CPC = cetylpyridinium chloride

Table 1 shows the basic characteristics of the patients. A total of 96 COVID-19-positive patients were enrolled from 7/11/2022, through 19/1/2023 (Fig. 1). Of these patients, 6 (6.3%) was excluded from analysis because they declined to participate after the allocation of mouthwash/placebo, which left 90 patients for inclusion in the primary analysis (Fig. 1). The median age of the patients at study entry was 33.1 years (interquartile range [IQR], 24.4–46.4), and 51 (56.7%) were male. Median body mass index was 22.4 (IQR, 20.3–24.3). The median number of days from the onset of symptoms to the initiation of the mouthwash trial was 2 (IQR, 1–2). The median pulse oximetry (SpO_2_) was 98 (IQR, 98–99). The median salivary Ct value at baseline was 38.0 (IQR, 36.5–39.8) in the placebo group, 38.6 (IQR, 35.3–41.2) in the CPC mouthwash group, and 35.2 (IQR, 32.6–38.1) in the on-demand aqueous chlorine dioxide mouthwash group.

**Table 1:** Baseline characteristics

		Placebo (*n* = 31)		CPC mouthwash (*n* = 31)		On-demand ACD mouthwash (*n* = 28)	
Male, n (%)		14	(45.2)	23	(74.2)	14	(50.0)
Median (IQR) age (years)		30.1	(23.1–47.8)	38.4	(25.5–47.4)	33.9	(27.1–40.5)
Median (IQR) body mass index (kg/m^2^)		22.5	(21.0–23.7)	22.3	(19.6–25.2)	22.2	(20.5–24.7)
Smoking history, n (%)							
	Non-smokers	20	(64.5)	21	(67.7)	22	(78.6)
	Former smokers	8	(25.8)	6	(19.4)	1	(3.6)
	Current smokers	3	(9.7)	4	(12.9)	5	(17.9)
Comorbidity (yes), n (%)		5	(16.1)	8	(25.8)	6	(21.4)
COVID-19 vaccination status, n (%)							
	None	1	(3.2)	0	(0.0)	1	(3.6)
	One dose	0	(0.0)	1	(3.2)	0	(0.0)
	Two doses	10	(32.3)	9	(29.0)	13	(46.4)
	Three doses	14	(45.2)	16	(51.6)	13	(46.4)
	Four or more doses	6	(19.4)	5	(16.1)	1	(3.6)
Median (IQR) days from onset to diagnosis		2	(1–2)	2	(1–2)	2	(1–2)
Median (IQR) pulse oximetry (SpO_2_)		99	(98–99)	98	(98–99)	98	(98–99)
Median (IQR) salivary Ct value		38.0	(36.5–39.8)	38.6	(35.3–41.2)	35.2	(32.6–38.1)

*Abbreviations* ACD = aqueous chlorine dioxide, CPC = cetylpyridinium chloride, Ct = cycle threshold, IQR = interquartile range

**Fig. 2:**
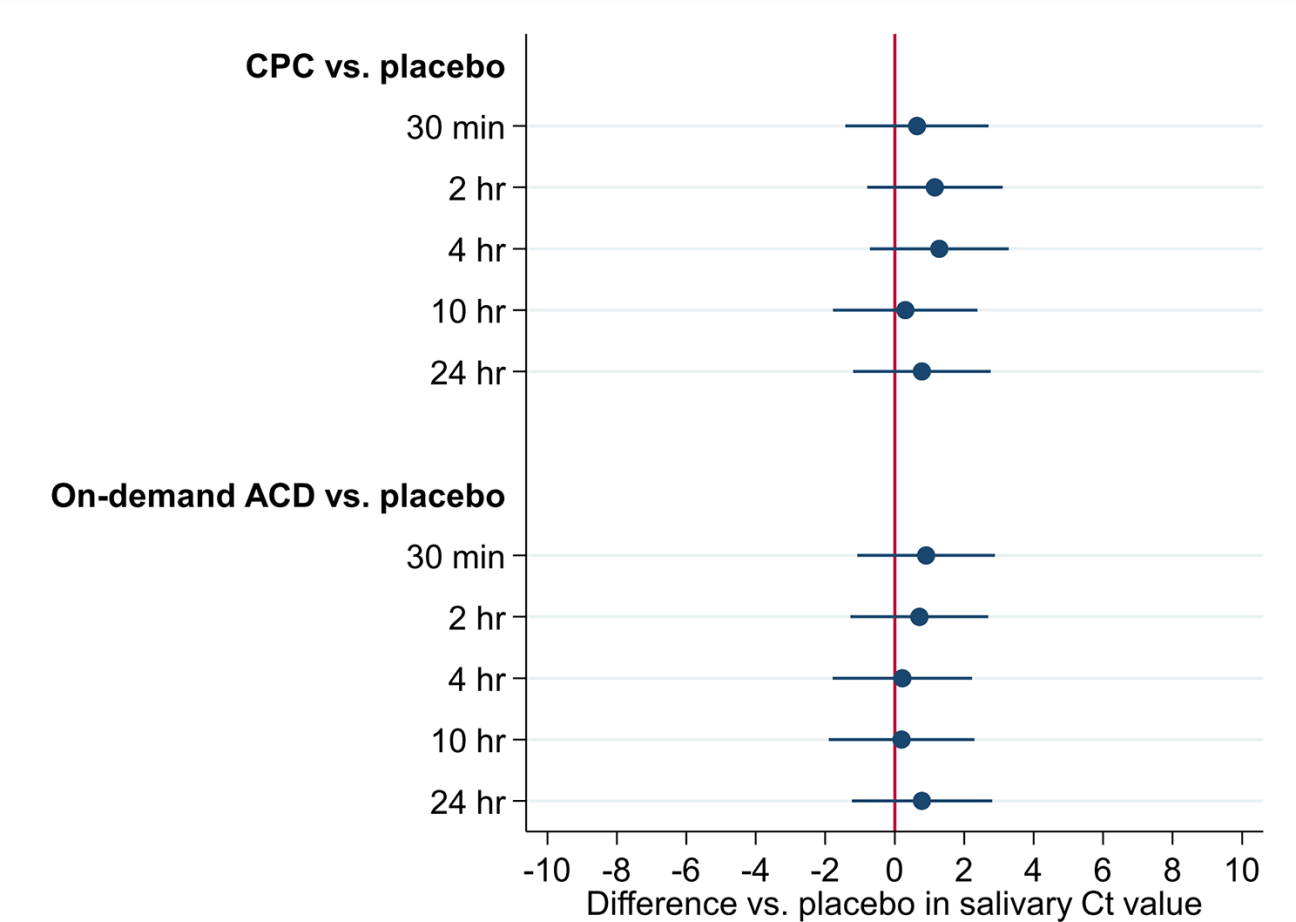
Difference vs. placebo in salivary Ct value. ACD = aqueous chlorine dioxide; CPC = cetylpyridinium chloride

The associations of 0.05% CPC and 0.01% on-demand aqueous chlorine dioxide mouthwash with salivary Ct values are shown in Table 2; Fig. 2. Compared with placebo, the use of 0.05% CPC mouthwash was not shown to be superior in changes from baseline salivary Ct values at 30 min (difference vs. placebo, 0.640; 95% confidence interval [CI], -1.425 to 2.706; *P* = 0.543); 2 h (difference vs. placebo, 1.158; 95% CI, -0.797 to 3.112; *P* = 0.246); 4 h (difference vs. placebo, 1.283; 95% CI, -0.719 to 3.285; *P* = 0.209); 10 h (difference vs. placebo, 0.304; 95% CI, -1.777 to 2.385; *P* = 0.775); or 24 h (difference vs. placebo, 0.782; 95% CI, -1.195 to 2.759; *P* = 0.438). The use of 0.01% on-demand aqueous chlorine dioxide mouthwash was also not shown to be superior to placebo in changes from baseline salivary Ct values at 30 min (difference vs. placebo, 0.905; 95% CI, -1.079 to 2.888; *P* = 0.371); 2 h (difference vs. placebo, 0.709; 95% CI, -1.275 to 2.693; *P* = 0.483); 4 h (difference vs. placebo, 0.220; 95% CI, -1.787 to 2.226; *P* = 0.830); 10 h (difference vs. placebo, 0.198; 95% CI, -1.901 to 2.296; *P* = 0.854); or 24 h (difference vs. placebo, 0.784; 95% CI, -1.236 to 2.804; *P* = 0.447).

**Table 2:** Difference vs. placebo in salivary Ct value

		Difference vs. placebo	95% CI		P value
CPC mouthwash vs. placebo					
	Baseline	Reference			
	30 min	0.640	(-1.425,	2.706)	0.543
	2 h	1.158	(-0.797,	3.112)	0.246
	4 h	1.283	(-0.719,	3.285)	0.209
	10 h	0.304	(-1.777,	2.385)	0.775
	24 h	0.782	(-1.195,	2.759)	0.438
On-demand ACD mouthwash vs. placebo					
	Baseline	Reference			
	30 min	0.905	(-1.079,	2.888)	0.371
	2 h	0.709	(-1.275,	2.693)	0.483
	4 h	0.220	(-1.787,	2.226)	0.830
	10 h	0.198	(-1.901,	2.296)	0.854
	24 h	0.784	(-1.236,	2.804)	0.447

*Abbreviations* ACD = aqueous chlorine dioxide, CI = confidence interval, CPC = cetylpyridinium chloride

In sensitivity analyses, the estimates showed little change following adjustment of baseline characteristics (Supplementary Table S1).

## Discussion

We investigated the effect of 0.05% CPC and 0.01% on-demand aqueous chlorine dioxide mouthwash compared with placebo on SARS-CoV-2 salivary viral load in asymptomatic to mildly symptomatic adults with COVID-19. Results showed that the use of 0.05% CPC and 0.01% on-demand aqueous chlorine dioxide mouthwash was not associated with a change from baseline SARS-CoV-2 salivary viral load as compared with placebo. Our study suggests that commercial 0.05% CPC and 0.01% on-demand aqueous chlorine dioxide mouthwash may not be useful in helping reduce or prevent infection with COVID-19. These results are consistent with a recent double-blind placebo-controlled randomized study, which provided the first clinical evidence that 0.07% CPC mouthwash did not reduce SARS-CoV-2 salivary viral load at 1–3 h after the intervention [14]. A recent multi-center, blind, parallel-group, placebo-controlled randomized clinical trial has also shown that no changes in salivary viral load of SARS-CoV-2 were observed after the use of 0.07% CPC mouthwashes at 30 min, 60 min, or 120 min [15]. A recent randomized, double-blind, controlled clinical trial has also indicated that SARS-CoV-2 RNA copy numbers were stable for up to 60 min after the use of 0.05% CPC mouthwash [16]. Recent systematic reviews have also shown that using 0.075% CPC-containing mouthwashes may not be effective in reducing SARS-CoV-2 viral load [40, 41]. This might be due to the fact that the antiviral effects of CPC against SARS-CoV-2 are the result of lipid membrane disruption in the viral envelope, without an effect on viral RNA integrity [42–44]. As soon as the lipid membrane is degraded, the viral capsid is exposed, leading to a decrease in the virus infectivity [16]. Additionally, it should be borne in mind that the oral clearance of food or drinks due to saliva swallowing may take up to half an hour for a person with healthy saliva flow; therefore, if viral particles are released from the oral mucosa and tongue tissues, a potential increase in viral load might be expected after using mouthwash [15]. In contrast, a recent experimental study in Japan has shown that 0.001–0.005% CPC in mouthwash suppressed the infectivity of human-isolated SARS-CoV-2 strains even in saliva without disrupting the viral envelope, and suggested that the anti-SARS-CoV-2 effects of CPC might not be due lipid membrane destruction but rather to the denaturation of SARS-CoV-2 protein [7]. Further evaluation of different concentrations of CPC and changes in levels of nucleocapsid protein associated with CPC mouthwash use is warranted.

Our findings also showed that 0.01% on-demand aqueous chlorine dioxide mouthwash may also not lead to a decrease in SARS-CoV-2 salivary viral load. To our knowledge, this study is the first RCT investigating the association of on-demand aqueous chlorine dioxide mouthwash with a reduction in salivary SARS-CoV-2 viral load. Our findings are inconsistent with a recent laboratory study that indicated that on-demand aqueous chlorine dioxide prevented the transmission of bacterial and viral infection via saliva [22]. Further RCTs with various concentrations and different specimens are warranted.

Our study may have practical implications. Antiviral drugs and vaccines may be insufficient to prevent the spread of COVID-19 infection, and public health services may need to prepare for COVID-19 infection through the implementation of a range of non-pharmaceutical preventive interventions. Mouthwashes are cheap, simple to use, and commonly employed as a daily antiseptic complement to brushing the teeth for oral hygiene maintenance [45]. Populations at high risk for contracting COVID-19 experience limited access to oral healthcare, and oral healthcare problems particularly affect poor and minority populations [46]. Thus, understanding the effects of physical interventions such as mouthwashes is important for planning public health policies for COVID-19. The results of our RCT showed that there were no clear differences between the use of CPC/on-demand aqueous chlorine dioxide mouthwash compared with placebo in reducing the SARS-CoV-2 salivary viral load. Several potential reasons for this can be proposed, including mouthwash concentrations and characteristics of the emerging SARS-CoV-2 variants. Viral load may vary with time and our results might change following the availability of further evidence with different concentrations of mouthwash from several brands or manufacturers and the continuous emergence of new SARS-CoV-2 variants. Additionally, sampling methods might also affect the effects of mouthwashes. Previous studies have indicated that supervised saliva collection may reduce variability between samples [47, 48]. Other studies also suggest that viral load is higher with throat gargle samples than with nasopharyngeal or oropharyngeal swabs [49, 50], and that saline mouth and throat gargle methods are more sensitive than saliva sampling [51]. Accurate evaluation of the efficacy of mouthwashes may require different sampling methods, such as the supervised collection of saliva samples, throat gargle sampling, nasopharyngeal swabs, and oropharyngeal swabs with multiple tests.

There are several limitations in this study. First, all enrolled patients had asymptomatic to mildly symptomatic COVID-19. Thus, our conclusion cannot be extrapolated to other clinical groups, including patients with moderate to severe cases of COVID-19 or patients with life-threatening disease. Additionally, key exclusion criteria included a previously confirmed COVID-19 infection with or without hospitalization. However, considering that study initiation was two years after the onset of the pandemic in 2020, patients may have been unknowingly exposed during this extended timeframe and the robustness of this criterion may not be assured. Second, our trial was not blinded to study group assignment. Thus, knowledge of the assignment of mouthwash/placebo and the extent of adherence with the intervention might have influenced the results. Third, our results might include potential biases because COVID-19 patients were recruited as volunteers; accordingly, the results might not be generalizable to the entire population. Fourth, because of difficulties in collecting data, we could not obtain information concerning individual factors such as income, education, employment, alcohol use, and nutrition. Although we did not observe remarkable differences in the baseline characteristics of the patients between groups in the use of mouthwash/placebo, other confounders may have influenced the results. Fifth, the RT-PCR method itself is limited in that it cannot differentiate between infective and non-infective virus. Although analytical testing with cultured virus be usable as a surrogate for clinical validity, it is unlikely to be a substitute for RT-PCR because of the advantages of RT-PCR in the early diagnosis of COVID-19 [52]. Additionally, patients 10 days out from onset with Ct values > 30 in saliva samples are less likely to infect others [53]. Thus, given the known difficulties of culturing the SARS-CoV-2 virus from clinical specimens, using viral RNA load as a surrogate is plausible [54], and the impact of this limitation in terms of clinical validity might be small. In contrast, RT-PCR might produce false negatives in the diagnosis [55], and the detection of infectious virus in collected samples is an important step in preventing COVID-19 infection. A previous study reported that the isolation efficiency of SARS-CoV-2 was significantly lower in saliva specimens than in the nasal/nasopharyngeal swab specimens [56]. To address the limitation of RT-PCR and enhance the accuracy of detecting infectious virus in collected samples, future studies of the efficacy of CPC and on-demand aqueous chlorine dioxide mouthwash on the viral viability of SARS-CoV-2 should be conducted using different specimen types and in multiple populations and settings.

In summary, our present trial suggests that 0.05% CPC and 0.01% on-demand aqueous chlorine dioxide mouthwash do not lead to a significant reduction in salivary SARS-CoV-2 viral load compared with placebo. Future studies with larger sample sizes and well-designed RCTs in multiple populations and settings are warranted.

### Electronic supplementary material

Below is the link to the electronic supplementary material.

Supplementary Material 1

## Data Availability

The datasets used and analyzed during the current study are available from the corresponding author on reasonable request.
