# Supplementary material for: Oral mouthwashes for asymptomatic to mildly symptomatic adults with COVID-19 and salivary viral load: a randomized, placebo-controlled, open-label clinical trial
Source: BMC Oral Health. 2024 Apr 25;24:491. doi: 10.1186/s12903-024-04246-1 (PMC11044332; doi:10.1186/s12903-024-04246-1)
Supplement: Supplementary file 1 — Supplementary Material 1 [file 12903_2024_4246_MOESM1_ESM.docx]

**Supplementary data**

**Table S1.** Sensitivity analysis, by adjusting for baseline characteristics (sex, age, body mass index, smoking history, comorbidity, COVID-19 vaccination status, days from onset to diagnosis, and pulse oximetry [SpO_2_]).

|  | | Difference vs. placebo | 95% CI | | P value |
| --- | --- | --- | --- | --- | --- |
| CPC mouthwash vs. placebo | |  |  |  |  |
|  | Baseline | Reference |  |  |  |
|  | 30 min | 0.698 | (-1.368, | 2.765) | 0.508 |
|  | 2 hr | 1.172 | (-0.782, | 3.127) | 0.240 |
|  | 4 hr | 1.304 | (-0.698, | 3.307) | 0.202 |
|  | 10 hr | 0.366 | (-1.715, | 2.447) | 0.730 |
|  | 24 hr | 0.787 | (-1.191, | 2.765) | 0.436 |
|  |  |  |  |  |  |
| On-demand ACD mouthwash vs. placebo | | | | | |
|  | Baseline | Reference |  |  |  |
|  | 30 min | 0.916 | (-1.067, | 2.899) | 0.365 |
|  | 2 hr | 0.738 | (-1.246, | 2.722) | 0.466 |
|  | 4 hr | 0.222 | (-1.784, | 2.229) | 0.828 |
|  | 10 hr | 0.247 | (-1.851, | 2.346) | 0.817 |
|  | 24 hr | 0.784 | (-1.235, | 2.804) | 0.446 |

Abbreviations: ACD = aqueous chlorine dioxide, CI = confidence interval, CPC = cetylpyridinium chloride.
